# Supplementary material for: The Crystal Structure of the Hsp90-LA1011 Complex and the Mechanism by Which LA1011 May Improve the Prognosis of Alzheimer’s Disease
Source: Biomolecules. 2023 Jun 28;13(7):1051. doi: 10.3390/biom13071051 (PMC10377191; doi:10.3390/biom13071051)
Supplement: Supplementary file 1 [file biomolecules-13-01051-s001.zip › biomolecules-2405726-supplementary (1).pdf]

Table S1. Summary of the ITC data for the binding interactions using Hsp90, FKBP51 and LA1011.

Cell component and concentration	Injectant and concentration	*K*d	N	Comment
30 μM Hsp90	400 μM FKBP51	-	-	One site fit. Overall, a poor fit
30 μM Hsp90	400 μM FKBP51	*K*d = 0.5 ± 0.1 μM<br>*K*d = 32.4 ± 6.3 μM	-	Two site fit
30 μM Hsp90	400 μM FKBP51-Δ7He	*K*d = 11.6 ± 1.1 μM	1.25	-
30 μM Hsp90	1 mM LA1011	*K*d = 13.1 ± 1.7 μM	0.5 (fixed)	-
30 μM Hsp90<br>60 μM FKBP51	1 mM LA1011	*K*d = 108 ± 3.0 μM	0.5 (fixed)	-
30 μM Hsp90<br>60 μM FKBP51-Δ7He	1 mM LA1011	*K*d = 17.9 ± 1.27 μM	0.5 (fixed)	-
30 μM Hsp90<br>1 mM LA1011	400 μM FKBP51	*K*d = 2.1 ± 0.2 μM	1.0	One site fit
30 μM Hsp90<br>1 mM LA1011	400 μM FKBP51	*K*d = 5.2 ± 2.7 μM<br>*K*d = 2.7 ± 1.9 μM	-	Two site fit
